# Viral kinetics in untreated versus treated acute HIV infection in prospective cohort studies in Thailand

**DOI:** 10.7448/IAS.20.1.21652

**Published:** 2017-06-26

**Authors:** Jintanat Ananworanich, Leigh Anne Eller, Suteeraporn Pinyakorn, Eugene Kroon, Somchai Sriplenchan, James LK Fletcher, Duanghathai Suttichom, Christopher Bryant, Rapee Trichavaroj, Peter Dawson, Nelson Michael, Nittaya Phanuphak, Merlin L Robb

**Affiliations:** ^a^ U.S. Military HIV Research Program, Walter Reed Army Institute of Research, Silver Spring, MD, USA; ^b^ Henry M. Jackson Foundation for the Advancement of Military Medicine, Bethesda, MD, USA; ^c^ SEARCH, The Thai Red Cross AIDS Research Centre, Bangkok, Thailand; ^d^ Department of Global Health, University of Amsterdam, Amsterdam, The Netherlands; ^e^ Armed Forces Research Institute of Medical Sciences, Bangkok, Thailand; ^f^ The EMMES Corporation, Rockville, MD, USA

**Keywords:** HIV, viral load, acute HIV, ART, early treatment, mathematical modelling

## Abstract

**Introduction**: The extent of viral replication during acute HIV infection (AHI) influences HIV disease progression. However, information comparing viral load (VL) kinetics with and without antiretroviral therapy (ART) in AHI is limited. The knowledge gained could inform preventive strategies aimed at reducing VL during AHI and therapeutic strategies to alter the viral kinetics that may enhance the likelihood of achieving HIV remission.

**Methods**: The analysis utilized VL data captured during the first year of HIV infection from two studies in Thailand: the RV217 study (untreated AHI, 30 participants and 412 visits) and the RV254 study (treated AHI, 235 participants and 2803 visits). Fiebig stages were I/II (HIV RNA+, HIV IgM−) and Fiebig III/IV (HIV IgM+, Western blot-/indeterminate). Data were modelled utilizing spline effects within a linear mixed model, with a random intercept and slope to allow for between-subject variability and adjustment for the differences in variability between studies. The number of knots in the quadratic spline basis functions was determined by comparing models with differing numbers of knots via the Akaike Information Criterion. Models were fit using PROC GLIMMIX in SAS v9.3.

**Results**: At enrolment, there were 24 Fiebig I/II and 6 Fiebig III/IV individuals in the untreated group and 137 Fiebig I/II and 98 Fiebig III/IV individuals in the treated group. Overall, the median age was 27.5 years old, most were male (89%), and CRF01_AE was the most common HIV clade (76%). By day 12 (4 days after ART in RV254), the untreated group had a 2.7-fold higher predicted mean VL level compared to those treated (predicted log VL 6.19 for RV217 and 5.76 for RV254, *p* = 0.05). These differences increased to 135-fold by day 30 (predicted log VL 4.89 for RV217 and 2.76 for RV254) and 1148-fold by day 120 (predicted log VL 4.68 for RV217 and 1.63 for RV254) (*p* < 0.0001 for both) until both curves were similarly flat at about day 150 (*p* = 0.17 between days 150 and 160). The VL trajectories were significantly different between Fiebig I/II and Fiebig III/IV participants when comparing the two groups and within the treated group (*p* < 0.001 for both).

**Conclusions**: Initiating ART in AHI dramatically changed the trajectory of VL very early in the course of infection that could have implications for reducing transmission potential and enhancing responses to future HIV remission strategies. There is an urgency of initiating ART when acute infection is identified. New and inexpensive strategies to engage and test individuals at high risk for HIV as well as immediate treatment access will be needed to improve the treatment of acute infection globally.

**Clinical Trial Number**: NCT00796146 and NCT00796263

## Introduction

The extent of viral replication during acute HIV infection (AHI) influences HIV disease progression. Most notably, viral set point levels in early infection predict time to clinical AIDS [1,2]. Recently, two prospective studies documented the kinetics of plasma viremia during acute infection in at-risk populations who underwent twice-weekly nucleic acid testing (NAT) [3,4]. Following the advent of viremia, the plasma viral load (VL) rises exponentially, typically reaching its peak within two weeks. This triggers HIV-specific CD8+ T cells that contributes to the initial control of viremia and the establishment of viral set point approximately four weeks later [5,6]. The set-point VL levels are associated with the magnitude and frequency of activated CD8+ T cells [4].

Antiretroviral therapy (ART) initiated in AHI rapidly lowers VL, reduces acute retroviral syndrome symptoms and mitigates CD4+ T cell depletion and immune dysfunction from HIV [7–9]. We and others have shown that viral suppression can be achieved expeditiously following early ART [10–12]. Time to virologic suppression appears shortest in people with lower baseline viremia such as those in early stages of acute infection prior to the appearance of HIV IgM (Fiebig I/II stages) when compared to later stages (Fiebig III/IV stages) [11,13]. However, there are little data on viral kinetics with and without treatment during Fiebig I–IV AHI.

With access to data from two unique AHI cohorts in Thailand, we performed statistical modelling of plasma VL kinetics of untreated vs. treated AHI amongst 265 individuals, during the first year of infection with a focus on the first 120 days of infection. The knowledge gained could inform preventive strategies aimed at reducing VL during AHI as well as therapeutic strategies given in addition to ART to alter the viral kinetics that may enhance the likelihood of achieving HIV remission.

## Methods

The analysis utilized data captured during the first year of infection from two ongoing studies in Thailand: the RV217 study (data from July 2009 to January 2012) and the RV254 study (data from April 2009 to April 2015). RV217 is a prospective cohort of individuals at high risk for HIV who are screened for AHI with twice-weekly NAT. ART is initiated according to standard of care at local health centres, and none in this analysis had ART. Some RV217 participants were in Fiebig III/IV at inclusion because of delay in returning to clinic for confirmatory testing. The RV254 cohort (NCT00796146) enrols individuals diagnosed with AHI at an HIV testing centre that has routine AHI screening, and they are offered immediate ART (NCT00796263). The RV254 participants included in this analysis initiated ART at a median of 2 (Inter Quartile Range (IQR) 1 to 3) days from enrolment. Fiebig stages at AHI diagnosis were categorized according to Fiebig and Busch: Fiebig I/II: HIV RNA+, p24 antigen±, HIV IgM− and Fiebig III/IV: HIV IgM+, Western blot-/indeterminate [13]. The follow-up assessments for VL in RV217 were on days 3, 7, 10, 14, 17, 21, 24, 28, 35, 42, 56 and 84 and then every three months. In RV254, these were performed on days 3, 5 and 7 and then at weeks 2, 4, 8, 12, 16, 20, 24, 36 and 48. All relevant ethics committees approved these studies, and each participant provided informed consent.

Because the RV217 had longitudinal and frequent sampling for NAT to diagnose AHI, the advent of viremia (i.e. the first detectable viremia) was able to be determined for RV217 participants. The RV254 study, in contrast, diagnosed AHI based on cross-sectional testing of samples from walk-in clinic clients; therefore, the advent of viremia could not be directly assessed. However, the pretreatment VL trajectories should be similar, on average, between the two cohorts. Thus, to ensure comparability of data, the time scale for RV254 participants was shifted as follows: Fiebig stage I/II individuals had their screening visit when AHI was diagnosed set to day 5 (from advent of viremia in RV217) and Fiebig stage III/IV individuals had their screening visit set to day 11. These shifts were determined by examining the mean VLs at screening in RV254 for each Fiebig stage subgroup and finding the corresponding points in the RV217 mean trajectory.

**Figure 1.**
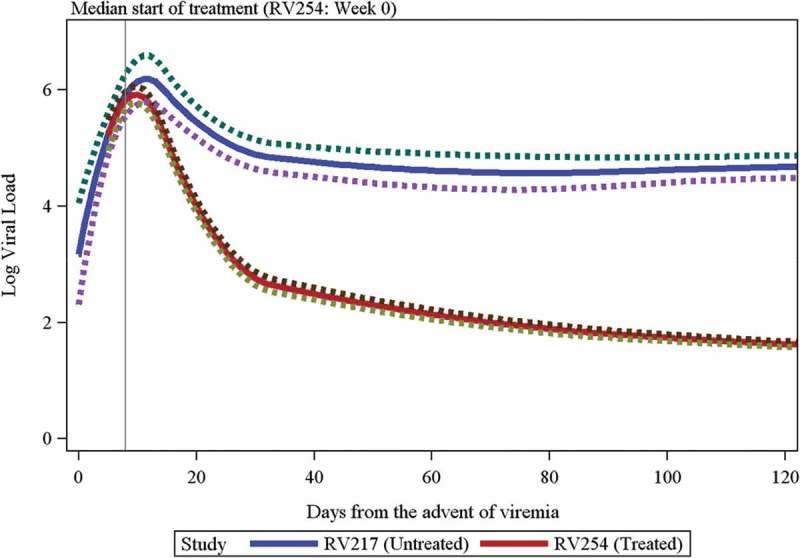
Predicted log viral load kinetics comparing RV217-untreated vs. RV254-treated acute HIV infection participants. The predicted values with a 95% confidence band are shown. The green and purple lines represent the upper and lower bounds of the 95% confidence intervals. The grey vertical line indicates the median start of treatment for RV254. Predicted log viral load means (95% confidence interval) for each study is given as follows: day 5 - RV217: 5.23 (4.87, 5.6), RV254: 5.21 (4.95, 5.48); day 10 - RV217: 6.14 (5.75, 6.54), RV254: 5.92 (5.78, 6.06); day 12 - RV217: 6.19 (5.78, 6.59), RV254: 5.76 (5.62, 5.9); day 30 - RV217: 4.89 (4.64, 5.14), RV254: 2.76 (2.64, 2.88); day 120 - RV217: 4.68 (4.48, 4.87), RV254: 1.62 (1.58, 1.67).

The choice of which day from the advent of viremia to assign to the screening visit in RV254 participants has a minimal impact on most of the estimates and hypothesis tests from the spline model shown in Figure 1 (see the results from sensitivity analyses in the Supplemental Tables 1 and 2). Shifting the Fiebig I/II screening visit in RV254 by four days instead of five leads to the difference in the study-specific trajectories between days 5 and 10 becoming significant at the *α* = 0.05 level. These sensitivity analyses indicate that the decisions made to align the time scales of the two studies are not driving the results of the analyses.

Forty-nine screening or baseline VL values for RV254 participants were above the assay’s upper limit of detection (LOD), and samples were unavailable for further dilution. For these observations, values were imputed as the mean of the observed RV254 VL values above the censoring threshold at the same visit. As a sensitivity analysis, these observations were dropped from the data set, and since this did not impact the inference or substantially alter the mean trajectory, the imputed values were included in the analyses. Additionally, 1216 values were censored at the highest lower LOD (50 copies/ml) and were imputed as LOD/2.

VL data from the two studies were then combined and modelled utilizing quadratic spline effects within a linear mixed model, with a random intercept and slope to allow for between-subject variability and adjustment for the differences in variability between studies (Supplemental Figure 1). Models with three to six knots, placed at the associated quantiles of the observed days from advent of viremia, were compared via the Akaike Information Criterion. The optimal model among these had four knots at the quintiles of days since the advent of viremia. Models were fit using PROC GLIMMIX in SAS v9.3.

## Results

A total of 265 Thai participants were included in the analyses: 30 from RV217 (24 Fiebig I/II and 6 Fiebig III/IV) and 235 from RV254 (137 Fiebig I/II and 98 Fiebig III/IV) (Table 1). The analysis contained 412 visits from RV217 and 2803 visits from RV254. Of the RV254 participants, 3 (1.3%) had a single pretreatment visit and 232 (98.7%) had two pretreatment visits; RV217 participants had a minimum of 4, a median of 16 and a maximum of 18 pretreatment visits. RV217 included younger participants (median age 23 vs. 26 years in RV254) and more transgender women (37% vs. 2% in RV254). Both groups displayed similar HIV subtypes (total group 76% CRF01_AE); however, RV217 participants had lower HIV RNA at first measurement (median of 4.4 vs. 5.7 log_10_ copies/ml in RV254) and greater CD4 counts (median of 707 vs. 371 cells/mm^3^ in RV254), likely due to differences in the baseline Fiebig stages. RV217 participants did not initiate ART after enrolment, whereas in RV254, 65% were on tenofovir/emtricitabine or lamivudine/efavirenz and 34% had maraviroc and raltegravir in addition to that regimen. The median (IQR) day from advent of viremia (after shifting the time scale as described previously) at the start of treatment for RV254 (i.e. week 0) was day 8 (7, 13). The loss-to-follow-up rate was low in the RV254 study: 1.7% (4/235) in this group and 3.7% (17/459) in the cohort in general. The loss of follow-up in the RV217 group reported here was 6.7% (2/30) and 28.5% (242/848) in the overall RV217 cohort in Thailand.

**Table 1.** Baseline characteristics of RV217-untreated and RV254-treated acute HIV infection participants

Characteristics	All	RV217 (untreated acute HIV)	RV254 (treated acute HIV)	*p*^d^
*N*	265	30	235	
Age, mean (SD)	27.5 (7.2)	23.8 (6.5)	28 (7.2)	0.0006
Gender, *n* (%)				<0.0001
Male	236 (89.1)	17 (56.7)	219 (93.2)	
Female	13 (4.9)	2 (6.7)	11 (4.7)	
Transgender	16 (6)	11 (36.7)	5 (2.1)	
Fiebig stage, *n* (%)				0.03
I/II (RNA+, p24 Ag±, HIV IgM−)	161 (60.8)	24 (80)	137 (58.3)	
III/IV (HIV IgM+, Western blot-/indeterminate)	104 (39.2)	6 (20)	98 (41.7)	
HIV subtypes, *n* (%)				0.58
CRF01_AE	202 (76.2)	23 (76.7)	179 (76.2)	
B	12 (4.5)	2 (6.7)	10 (4.3)	
CRF01_AE/B recombinant	23 (8.7)	1 (3.3)	22 (9.4)	
Others	28 (10.6)	4 (13.3)	24 (10.2)	
HIV RNA^a^ (log_10_copies/ml)				0.0003
Mean (SD)	5.3 (1.6)	4.2 (1.8)	5.5 (1.5)	
Median (IQR)	5.6 (4.4, 6.6)	4.4 (2.7, 5.9)	5.7 (4.5, 6.7)	
CD4 T cells^b^ (cells/mm^3^)				<0.0001
Mean (SD)	436.5 (234.7)	777.4 (349)	400 (186)	
Median (IQR)	393.5 (276, 531)	707 (473, 993)	371 (269, 504)	
CD4 T cells/CD8 T cells				**0.009**
Mean (SD) Median (IQR)	0.88 (0.54) 0.79 (0.47, 1.17)	1.21 (0.68) 1.19 (0.62, 1.74)	0.85 (0.52) 0.74 (0.47, 1.13)	
ART regimens initiated after enrolment^c^, *n* (%)		Not applicable		
TDF/XTC/EFV	152 (57.4)		152 (64.7)	
	80 (30.2)		80 (34)	
TDF/XTC/EFV/MVC/RAL Other	3 (1.1)		3 (1.3)	

^a^First available viral load measurement for RV217 participants and screening viral load for RV254 participants.

^b^First available CD4 count for RV217 participants and week 0 CD4 count for RV254 participants.

^c^First ART regimen initiated.

^d^Wilcoxon rank-sum test used for continuous variables and Fisher’s exact test used for categorical variables.

TDF: tenofovir; XTC: lamivudine or emtricitabine; EFV: efavirenz; MVC: maraviroc; RAL: raltegravir; ART: antiretroviral therapy; SD: standard deviation; IQR: Inter Quartile Range.

The predicted VL values with 95% confidence bands are shown through day 120 in Figure 1. The trajectory over time was significantly different between the two studies (*p* < 0.001). The initial rise in VL was similar between the two studies (*p* = 0.34 for testing the difference in slopes between days 5 and 10), but after the RV254 peak at about 10 days, the trajectories differed (*p* < 0.001 for testing the difference in slopes between days 10 and 12, the latter of which was the predicted peak for RV217 participants; *p* < 0.001 between days 12 and 28; *p* < 0.001 between days 28 and 42) until both curves were similarly flat at about day 150 (*p* = 0.17 between days 150 and 160). At day 10, the predicted mean VL levels were similar between RV217 and RV254 with a fold change of 1.7 (predicted log VL 6.15 for RV217 and 5.92 for RV254; *p* = 0.29). By day 12, however, the RV217 cohort had a 2.7-fold higher predicted mean VL level compared to RV254 (predicted log VLs 6.19 for RV217 and 5.76 for RV254; *p* = 0.05). These differences increased to 135-fold by day 30 (predicted log VLs 4.89 for RV217 and 2.76 for RV254) and 1148-fold by day 120 (predicted log VLs 4.68 for RV217 and 1.62 for RV254) (*p* < 0.0001 for both).

**Figure 2.**
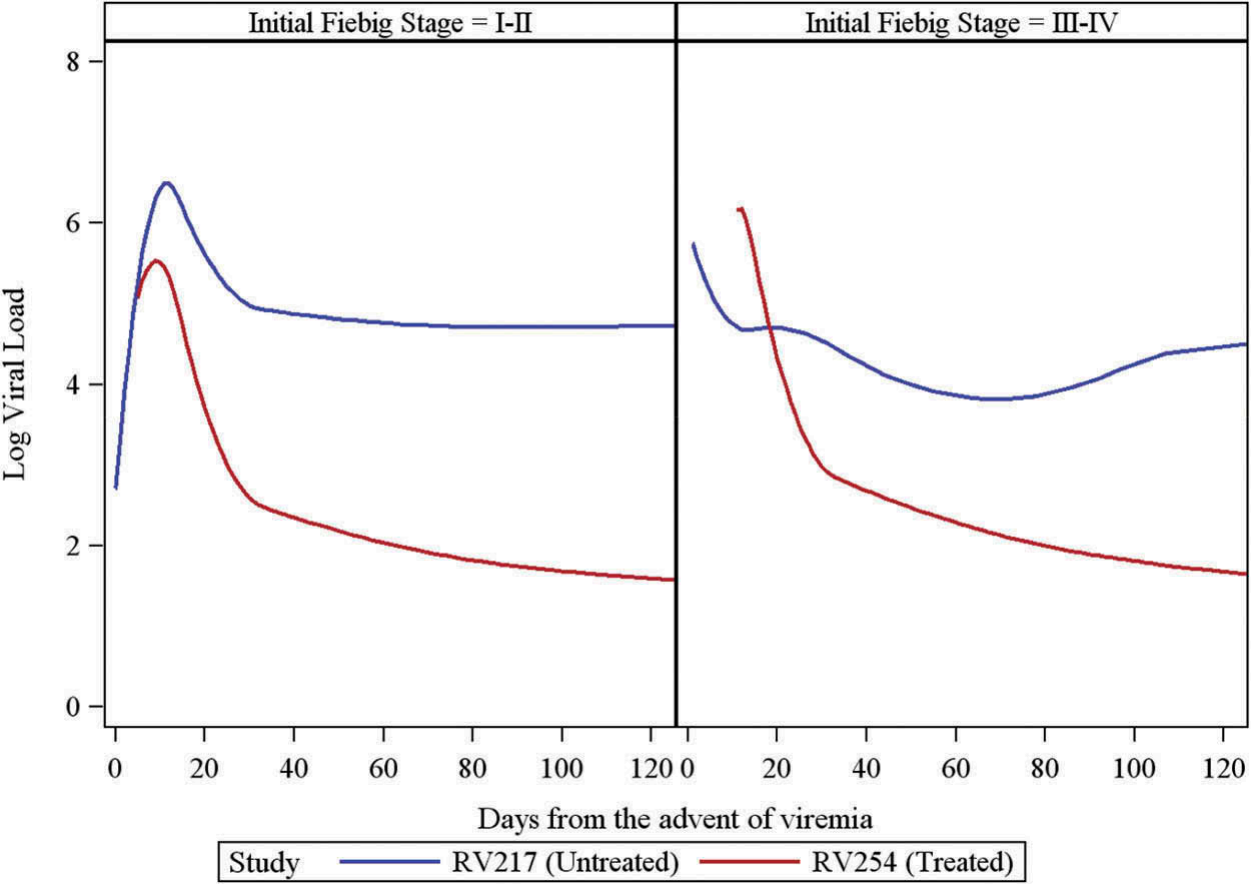
Predicted log viral load values from a spline model within each Fiebig stage comparing RV217-untreated vs. RV254-treated acutely infected individuals. Fiebig I/II: HIV RNA+, p24 Ag+/−, HIV IgM−; Fiebig III/IV: HIV IgM+, Western blot-/indeterminate.

Observed peak viremia occurred at a median (IQR) of 13.5 (9–15) days in RV217 and 10 (7–11) days in RV254 (on the shifted time scale), yielding a *p* value of 0.001 from a Wilcoxon rank-sum test. The true peak viremia was only observed for RV217 participants, since in the RV254 cohort treatment could have been initiated before the peak was reached in some participants. Peak VL levels did not differ significantly between cohorts (medians of 6.5 vs. 6.2 log_10_ copies/ml in RV217 and RV254, respectively, Wilcoxon *p*-value = 0.09). However, within Fiebig I/II, peak VL was significantly higher in RV217 compared to RV254 (medians of 6.67 and 5.83 log_10_ copies/ml in RV217 and RV254, respectively, *p* < 0.001), but within Fiebig III/IV, peak VL was significantly lower in RV217 compared to RV254 (medians of 5.46 and 6.6 log_10_ copies/ml in RV217 and RV254, respectively, *p* = 0.02). The latter could be attributed to the small sample size with only six RV217 participants in Fiebig III/IV. Additionally, peak VL likely occurred before entry into the study for some RV254 Fiebig III/IV participants. In a separate model, different trajectories were fit for each Fiebig stage subgroup and study, with predicted values shown in Figure 2. The differences between RV254 and RV217 trajectories significantly differed by initial Fiebig stage (*p* < 0.001), but due to the small number of Fiebig III/IV among RV217 participants, interpretation is difficult. Among RV217 participants entering the study as Fiebig I/II, the predicted doubling time from the spline model was approximately 0.6 days. Additionally, within the RV254 cohort only, the VL trajectories were significantly different between Fiebig I/II and Fiebig III/IV participants (chi-squared test: *p* < 0.001). Also, mean VL differed between treated and untreated RV254 visits (2123 treated visits and 680 untreated visits, including pretreatment phase and gaps between regimens), after adjusting the time from the advent of viremia (*p* < 0.001). The VL trajectories between the stages were not statistically different in RV217.

**Figure 3.**
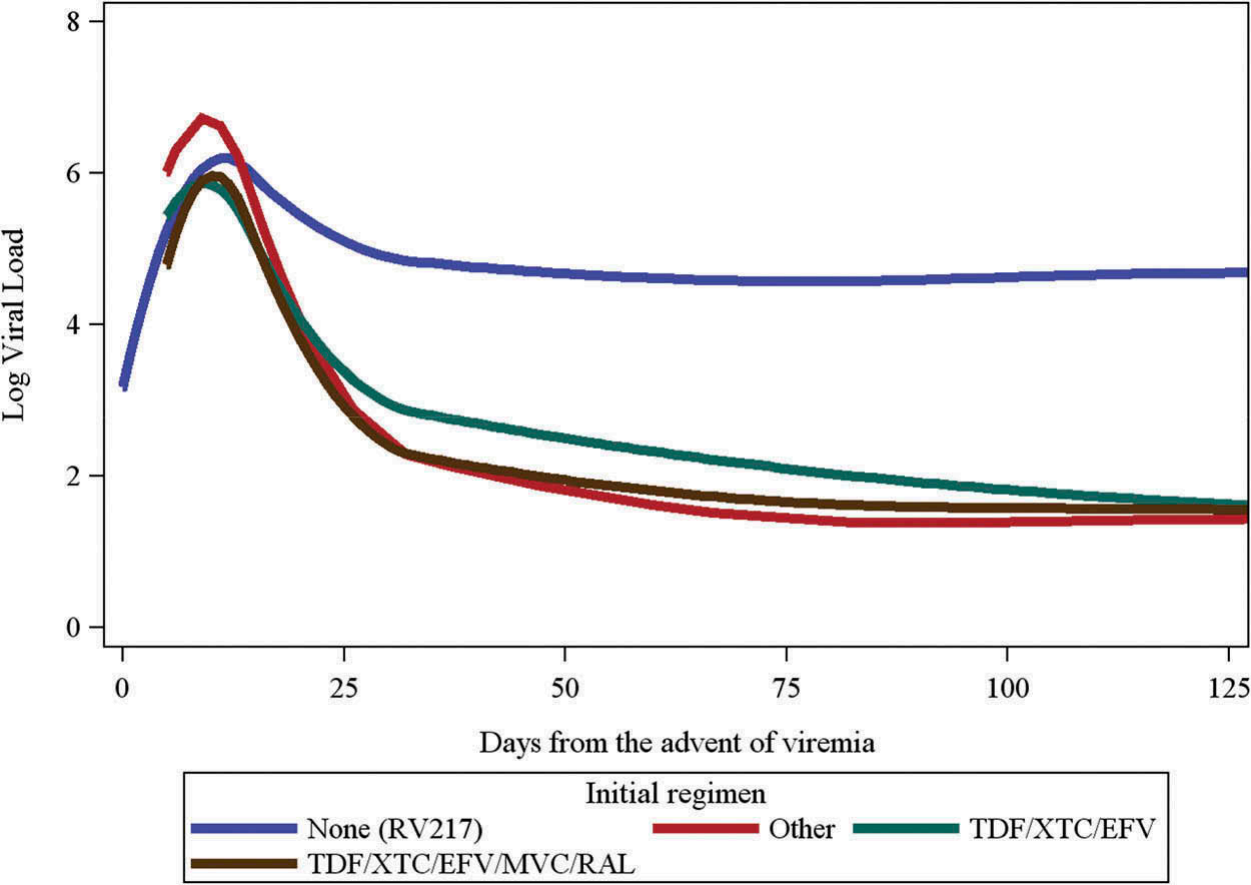
Predicted log viral load trajectories by antiretroviral regimen. Number (%) of participants in RV254 by regimens were 152 (57.4%) for TDF/XTC/EFV, 80 (30.2%) for TDF/XTC/EFV/MVC/RAL and 3 (1.1%) for other regimens.TDF: tenofovir; XTC: lamivudine or emtricitabine; EFV: efavirenz; MVC: maraviroc; RAL: raltegravir.

HIV subtype and gender or risk group did not significantly affect VL levels and trajectories across the studies. In RV254, treatment with raltegravir (*n* = 80) resulted in faster VL decline with lower log VLs at day 25 (TDF/XTC/EFV predicted log VL 3.38) and day 100 (TDF/XTC/EFV predicted log VL 1.82) compared to regimens without raltegravir (*n* = 155) (TDF/XTC/EFV/MVC/RAL predicted log VLs 2.92 and 1.58 on day 25 and day 100, respectively, *p* < 0.001 for both comparisons, Figure 3 - the group without treatment is the RV217 cohort).

## Discussion

Utilizing statistical modelling of VL kinetics based on one of the largest data sets from acutely infected participants, we observed that initiating ART in acute infection dramatically changed the trajectory of VL very early in the course of infection. Beginning 12 days from the advent of viremia or approximately 4 days after ART initiation, a 2.7-fold difference of VL means was predicted with the gap widening to 1148-fold by day 120. Peak viremia was also higher in untreated Fiebig I/II vs. those treated. These observations highlight the urgency of initiating ART as early as possible when acute infection is diagnosed.

The VL kinetics observed in our studies are similar to the earlier acute simian immunodeficiency virus and HIV studies, which showed that VL levels and patterns were influenced by the available target cells and host immune responses [14–16]. The levels of viremia during the first week of acute infection and at set-point were highly correlated; the latter was a critical determinant for disease progression [1,15]. The decline from peak viremia was due mainly to the depletion of productively infected cells [14–16]. Recently, two studies prospectively screened at-risk individuals for AHI and carefully documented viral kinetics during this period: the FRESH cohort of young South African females [4] and our RV217 cohort in East Africa (predominantly female participants) and Thailand (predominantly male participants) [3]. The median plasma VL at first detection was about 3–4 log_10_ copies/ml and within two weeks rose to 6–7 log_10_ copies/ml. We observed a VL doubling time of 0.6 log 10 per day in the absence of treatment similar to those in the South African and US cohorts [3,4,14,17]. There is, however, some variability in VL levels between populations, with higher levels observed in the Africans and Thais than among Americans [3,4,14,17]. These differences in VL kinetics between populations could be from sex, region, HIV clades, host factors, immunologic responses, timing of samples and inter-person variability. A swift and strong CD8+ T cell response can lower viral set point [4,18].

HIV replication is halted soon after ART initiation, resulting in an early divergence of VL trajectories between the treated and untreated individuals. Inhibiting viral replication prevents viral mutation development and reduces immune destruction, reservoir seeding and immune activation [19–25]. This provides a strong rationale for studying immunotherapeutics in early treated individuals who have little or no viral escape, less CD4 depletion and more preserved memory T cells in order to improve HIV-specific immune responses and possibly enhancing the chance for HIV remission (i.e. undetectable plasma VL levels following ART discontinuation) [26–28]. High VL during acute infection increases the risk for onward transmission [24,29–32]; therefore, modifying the trajectory of VL with early ART could have an important public health implication for HIV prevention. The shorter time to viral suppression with integrase inhibitor-based regimens during acute infection may bear additional preventive and therapeutic benefits [23].

The strength of our work is the inclusion of data from a relatively rare population of individuals in early HIV infection with both people who initiated ART early vs. those who did not. The population is homogenous: they are all Thais with fairly comparable age, sex and HIV subtypes. The main limitation is that time from advent of viremia was determined only for RV217 requiring a shift in the time scale for RV254 participants to align the untreated portions of both cohorts’ VL trajectories. This approach gives only a rough estimate of an RV254 participant’s entry into the study relative to the advent of viremia. Given the limitations of the available data, though it appears to do well in capturing the mean trajectory. Additionally, the number of RV217 participants in Fiebig III/IV was small and limits the comparisons within the same study and across the two studies.

## Conclusions

ART in acute infection significantly altered the trajectory of VL from day 4 after ART initiation, with the VL differences between the untreated and treated cohorts widening to 1148-fold by day 120. Altering the VL trajectory could have implications for reducing transmission potential and enhancing responses to future HIV remission strategies. These observations highlight the urgency of initiating ART when acute infection is diagnosed. Identifying acutely infected individuals is challenging. Innovative strategies will be needed to improve the detection and treatment of acute infection globally.
